# The Complete Chloroplast Genome of An *Ophiorrhiza baviensis* Drake Species Reveals Its Molecular Structure, Comparative, and Phylogenetic Relationships

**DOI:** 10.3390/genes14010227

**Published:** 2023-01-15

**Authors:** Mai Huong Pham, Thu Hoai Tran, Thi Dung Le, Tung Lam Le, Ha Hoang, Hoang Ha Chu

**Affiliations:** 1 Institute of Biotechnology (IBT), Vietnam Academy of Science & Technology (VAST), Hanoi 100000, Vietnam; 2 Faculty of Biotechnology, Graduate University of Science and Technology, VAST, Hanoi 100000, Vietnam

**Keywords:** *Ophiorrhiza baviensis*, chloroplast genome, comparative analysis, phylogeny

## Abstract

*Ophiorrhiza baviensis* Drake, a flowering medical plant in the Rubiaceae, exists uncertainly within the *Ophiorrhiza* genus’ evolutionary relationships. For the first time, the whole chloroplast (cp) genome of an *O. baviensis* Drake species was sequenced and annotated. Our findings demonstrate that the complete cp genome of *O. baviensis* is 154,770 bp in size, encoding a total of 128 genes, including 87 protein-coding genes, 8 rRNAs, and 33 tRNAs. A total of 59 SSRs were screened in the studied cp genome, along with six highly variable loci, which can be applied to generate significant molecular markers for the *Ophiorrhiza* genus. The comparative analysis of the *O. baviensis* cp genome with two published others of the *Ophiorrhiza* genus revealed a high similarity; however, there were some notable gene rearrangements in the *O. densa* plastome. The maximum likelihood phylogenetic trees were constructed based on the concatenation of the *rps16* gene and the *trnL-trnF* intergenic spacer sequence, indicating a close relationship between the studied *O. baviensis* and other *Ophiorrhiza*. This study will provide a theoretical molecular basis for identifying *O. baviensis* Drake, as well as species of the *Ophiorrhiza* genus, and contribute to shedding light on the chloroplast genome evolution of Rubiaceae.

## 1. Introduction

The chloroplast (cp) is an essential organelle in photosynthetic plant and microbial cells that produces energy to feed the cell through photosynthesis [1]. Each chloroplast contains its own ribosomes and a separate genome from the cell’s nuclear genome, ranging in size from 20 to 160 kilobase pairs (kp). The cp genome is uniparentally inherited with a quadripartite structure consisting of one large single-copy (LSC) region, one small single-copy (SSC) region, and two inverted repeat regions (IRs) of the same length [2]. As a result of the small size of the cp genome, which contains only around 100 to 120 protein coding genes, chloroplasts are often the first target for sequencing in evolutionary analysis, barcoding, and meta-barcoding [2]. In the NCBI Genbank database at present, there are more than 1000 cp genomes of plant species. However, this number is very small compared to the existing plant diversity on the planet, which raises the need to collect and store sequences of uncharacterized species. 

For medicinal plants such as *Ophiorrhiza baviensis*, the potential for exploitation and the need for systematic classification are even more essential. *O. baviensis* is a species of flowering plant in the Rubiaceae family, first described scientifically by Drake in 1895, and re-identified by Wu et al. [3]. Information on the ecology and genomic characteristics of this species is extremely limited, with only four sequences of *O. baviensis*—the gene junctions *trnL-trnF* (#MH626989.1), *rps16* (#MH626923.1), the external transcribed spacer (ETS) (#MH626743.1), and *ITS* (#MH626804.1)—available on the Genbank database of the National Center for Biotechnology Information (USA) (NCBI). Each sequence is less than 1000 base pairs (bp) in size, only two of which belong to the chloroplast genome. Thus, there is a need to study the entire chloroplast genome of *O. baviensis* species for taxonomy and diversity assessment, as well as chloroplast genome characterization, conservation, and future research. With an estimated chloroplast genome size of 154 kb, the potential for exploiting genomic information on the *O. baviensis* chloroplast genome is very large.

Recently, PacBio sequencing technology has been applied to sequence cp genomes, and there have been studies demonstrating the superior ability of PacBio in de novo assembly with 99% accuracy; moreover, as the repeatability increases, this can exceed 99.9% [4]. PacBio sequencing is also a great technology in resolving gaps in rRNA, i.e., internal transcribed spacer (ITS) regions and the surrounding regions to obtain accurate molecular biology information for species identification. For the first time, we report a new complete chloroplast genome of *O. Baviensis* Drake from Vietnam and compare it with previously published *Ophiorrhiza* complete chloroplast genome data to evaluate the genome organization, phylogenetic relationships, and conserved genetic resources.

## 2. Materials and Methods

### 2.1. Sample Collection and Chloroplast Genome Sequencing

**Figure 1:**
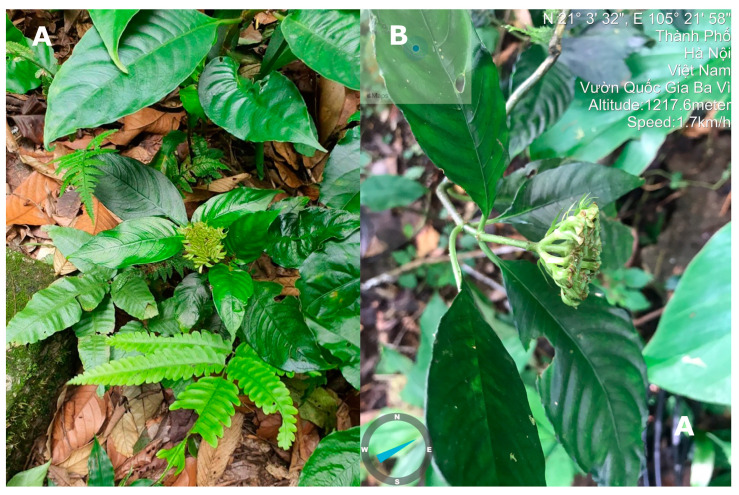
*O. baviensis* Drake. (**A**) Habitat; (**B**) Morphological characteristic of infructescence in side view; Photos by Thu Hoai Tran.

*O. baviensis* samples were collected in Ba Vi National Park, Hanoi, Vietnam in August 2022 (code number: Xacan 01), 1217.6 m, 21°3′32″ N; 105°4′58″ E (Figure 1). The voucher specimens were placed in the herbarium of the Institute of Ecology and Biological Resources (HN), Hanoi, Vietnam. Fresh leaves with the same code number were used to extract genomic DNA.

### 2.2. DNA Extraction and Chloroplast Genome Sequencing

We treated samples prior to extraction with the Chloroplast Isolation Kit (ab234623-Abcam, Cambridge, UK) for cp enrichment to increase the cpDNA concentration. The total DNA was extracted by the GeneAll® Exgene™ Plant SV mini kit using the enriched samples (including both genomic DNA and cp DNA). The extracted DNA integrity was evaluated by electrophoresis on a 0.8% agarose gel for 45 minutes at 120 V, and the DNA concentration was measured by Nanodrop 2000 (Thermo, Waltham, MA, USA) and Qubit 2.0 devices to ensure quality for library preparation and sequencing on the Pacbio system according to the manufacturer’s instructions.

The total DNA was fragmented and the DNA damage from fragmentation, as well as the 5′/3′ ends, underwent repair using the SMRTbell Damage Repair Kit SPv3 (#100-992- 200, Pacific Biosciences, Menlo Park, CA, USA) before being attached to PacBio adapters. Products without adapters are rejected by the Exo III and Exo VII enzymes. The SMRTbell library was cleaned with Ampure PB beads (Beckman Coulter, Brea, CA, USA) and checked for length and concentration using the Bioanalyzer 2100. Subsequently, it was cleaned and sized using BluePippin (SageScience, Beverly, MA, USA) with a gel concentration of 0.75% to filter out library DNA fragments above 20 kb in length. The library was lastly checked for size and fragmentation with the Bioanalyzer 2100 before loading to the SMRT Cell (#101-008-000, PacBio).

The prepared library was loaded on one chip and sequenced on a PacBio SEQUEL system at the National Key Laboratory for Gene Technology, Institution of Biotechnology (Hanoi, Vietnam). SMRTbell library was attached with polymerase and purified using the Sequel Binding Internal Ctrl Kit 2.0 (#101-400-900, PacBio) and the SMRTbell Clean Up Column v2 Kit-Dif (101-184-100, PacBio) according to the procedure generated by the Sample Setup software included in the SMRTLink portal version 5.1.

### 2.3. Genome Assembly and Annotation

Total DNA was sequenced using the PacBio platform. Sequences derived from the cp genome were identified through the pbmm2 program using the cp genome of the reference *Ophiorrhiza* species (accession number: NC_057496.1) obtained from the Genbank database [5]. Then, the Hierarchical Genome Assembly Process version 4 (HGAP4) software was used to assemble the cp genome [6]. Protein-coding genes and RNA were annotated by the GeSeq webtool [7], while tRNAscan-SE software version 2.0 was applied to verify the tRNA genes [8]. The OrganellarGenomeDRAW (OGDRAW) web-tool was selected to generate the circular gene map [9]. Repeat elements were identified using two approaches. The web-based MISA finder was used for detecting microsatellites in nucleotide sequences, with the following parameters: 10 repeats for mono-, 5 for di-, 4 for tri-, and 3 for tetra-, penta-, and hexa-nucleotide SSRs [10]. Size comparison of the SSRs among the SSRs of each type was used to count polymorphic SSRs. The size and pattern of repeats in the cp genome were identified using the REPuter with the following set of parameters: minimum repeat size 20 bp, hamming distance 3 kb, and 90% or more sequence similarity [11].

### 2.4. Genome Comparison and Phylogenetic Identification

For cp genome comparison, we collected available cp genomes of *Ophiorrhiza* species (*O. pumila*—NC_057496.1 and *O. densa*—NC_058252.1) from the GenBank database (https://www.ncbi.nlm.nih.gov/genbank/, accessed on 15 November 2022). The overall genome structure, gene content, genome size, and number of repeats across the genomes were compared. The entire cp genome sequences of the *Ophiorrhiza* species were aligned through MAFFT software with default parameters and visualized in the mVISTA webtool with the LAGAN mode [12]. We used the annotated cp genome of the project as the reference genome in the mVISTA diagram. Subsequently, Irscope was used to visualize and compare the contiguous region between the large and small single-copy, along with the inverted repeat regions of the genomes. We also examined codon usage bias and sequence divergence via computational nucleotide diversity (Pi) analysis among cp genomes in DnaSP software version 6.12.03 [13]. For the sequence divergence analysis, we applied a window size of 600 bp with a step size of 200 bp.

A concatenation of the *rps16* gene and *trnL-trnF* intergenic spacer sequences from the *Ophiorrhiza* species and two *Xanthophytum* species of the Rubiaceae family from the Genbank database was used to identify the phylogenetic relationships of the studied *O. Baviensis* Drake. The nucleotide sequences were aligned with MAFFT software with default parameters [14] before the maximum likelihood (GTR+CAT model) phylogenetic tree was constructed using FastTree [15] with a 1000 bootstrap and visualized by FigTree software version 1.4.4 (http://tree.bio.ed.ac.uk/software/figtree/, accessed on 1 July 2021).

## 3. Results

### 3.1. Chloroplast Genome Assembly and Annotation

**Figure 2:**
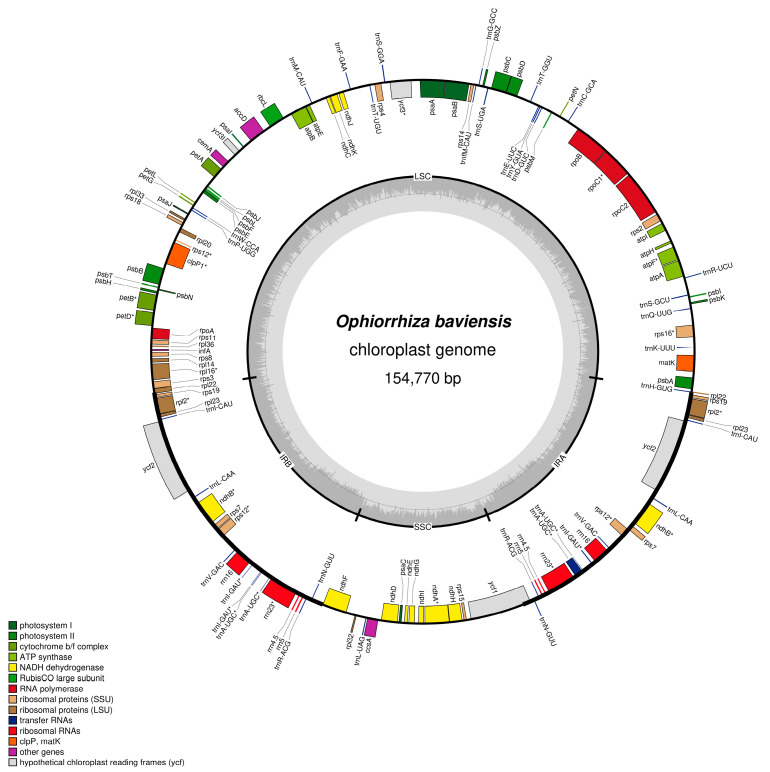
Chloroplast genome map of *O. baviensis* Drake in Vietnam. Genes shown inside the circle are transcribed clockwise, whereas genes outside are transcribed counterclockwise. The light gray inner circle shows the AT content, the dark gray corresponds to the GC content.

Using the PacBio SEQUEL I system, 28,402,467,862 bp of raw sequence data were generated with a mean read length of 1938 bp, an N50 contig size of 2412 bp, and approximately 9% of the raw reads belonging to the *O. baviensis* cp genome with 158 × coverage. The resequencing assembly resulted in a circular cp genome size of 154,770 bp (Figure 2), and the percentage of GC content was 37.6%. As reported in most angiosperm cp genomes, the assembled *O. baviensis* Drake plastome demonstrated the typical quadripartite structure consisting of four regions, LSC (84,626 bp), SSC (18,574 bp), and a pair of inverted repeats (IRs 25,685 bp). 

In addition, the annotation results from GeSeq and tRNAscan-SE revealed that the *O. baviensis* Drake cp genome possessed a total of 128 genes, of which there were 87 protein-coding genes, 33 tRNA genes, and 8 rRNA genes (16S, 23S, 5S, and 4.5S) (Table 1). The annotated gene models were assigned into three major groups based on their functions. Regarding the photosynthesis-related gene category, there were 44 genes encoding the subunits of ATP synthase, cytochrome complex, photosystem I and II, and putative NADPH dehydrogenase, along with the large subunit of Rubisco related to the photosynthetic electron transport chain. The other 76 genes were functionally characterized in the transcription and translation processes. The majority were tRNA genes, and the others were rRNA genes and genes encoding DNA-dependent RNA polymerase, the subunits of the ribosome, and ribosome proteins. The remaining nine genes were classified in the category of other genes, consisting of five genes with reported functions in RNA processing (*matK*), c-type cytochrome synthesis (*ccsA*), fatty acid synthesis (*accD*), carbon metabolism (*cemA*), and proteolysis (*clpP*). In addition, four genes encoding the conserved reading frames (*ycf1, ycf2, and ycf3*) were also annotated in the cp genome. 

Otherwise, each IR region of the *O. baviensis* cp genome was annotated to comprise 18 genes (all 4 rRNA genes, 7 tRNA genes, 1 NADH-dehydrogenase protein-coding gene, 4 ribosomal protein-coding genes, and 2 other genes). There were 17 cp genes that harbored introns, among which 15 genes (*atpF*, *rpl2* (×2), *rpl16*, *ndhA*, *ndhB* (×2), *rpoC1*, *rps12*, *rps16, trnA-UGC* (×2), *trnG-GCC*, and *trnI-GAU* (×2)) contained a single intron, while two genes (*ycf3*, *clpP*) had double introns (Table 2).

### 3.2. Repeat Sequences and Codon Analysis

**Figure 3:**
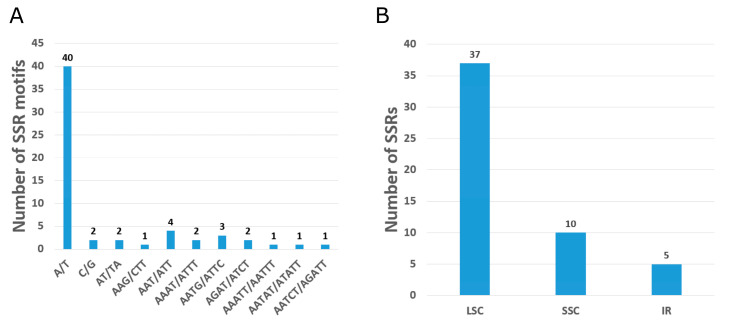
Analysis of single sequence repeats (SSRs) of the *O. baviensis* Drake chloroplast genome. (**A**) Number of identified SSR sequence motifs; (**B**) Frequency of repeat types in LSC, SSC, and IR regions.

A total of 59 simple sequence repeats (SSRs) were investigated in the *O. baviensis* Drake chloroplast genome via the MISA web-tool. Almost all of the screened repeats were mono repeats (composed of A/T and C) with the size ranging from 10 to 16 bp (Figure 3A). Two di-, five tri-, seven tetra-, and three penta-nucleotide SSRs were found in the *O. baviensis* Drake plastid. A total of 53 SSRs were classified as simple based SSRs and the six remaining SSRs presented in a compound formation. The majority of SSR types were discovered in the LSC, while the IR regions included the smallest number of SSRs (Figure 3B).

**Figure 4:**
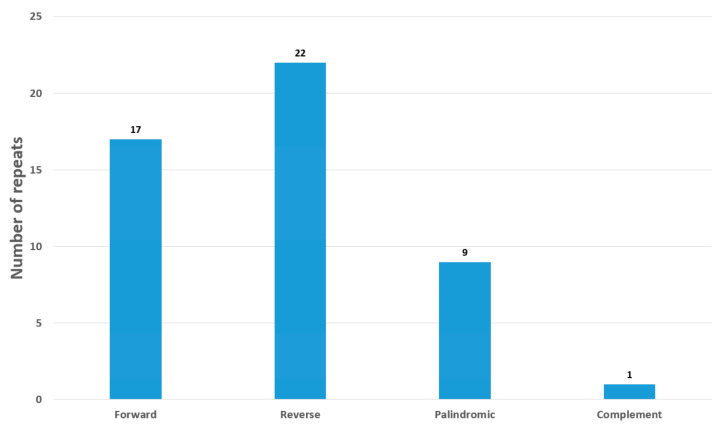
Repeat analysis of *O*. *baviensis* Drake chloroplast genome.

The cp genome of *O. baviensis* Drake was annotated to possess 49 long repeats including 9 palindromic repeats, along with 12 forward and 22 reverse repeats. There was only one complement repeat (Figure 4). The unit size of the detected repeats ranged from 20 to 58 bp, while a majority of the repeat size (67%) was shorter than 30 bp. 

The codon usage frequency of 64 protein-coding genes was evaluated for three cp genomes: *O. baviensis* Drake and two other available *Ophiorrhiza* species. The total number of codons found in the coding regions was 51,517, while the A- and U-ending were found more frequently than the G/C-ending (Table 3). Leucine was the most prevalent among the 20 amino acids with a percentage of 10.46% (5068 codons), followed by serine with 9.95% (4817 codons). Meanwhile, the rarest was tryptophan with a total of 681 codons accounting for approximately 1.4%. A total of 30 codons exhibited the codon usage bias (RSCU < 1), while 32 codons were observed to be more frequent than the expected usage at equilibrium (RSCU > 1) (Table 3). The usage frequency for the start codons AUG and UGG (methionine and tryptophan) exhibited no bias (RSCU = 1).

### 3.3. Chloroplast Genome Comparison

**Figure 5:**
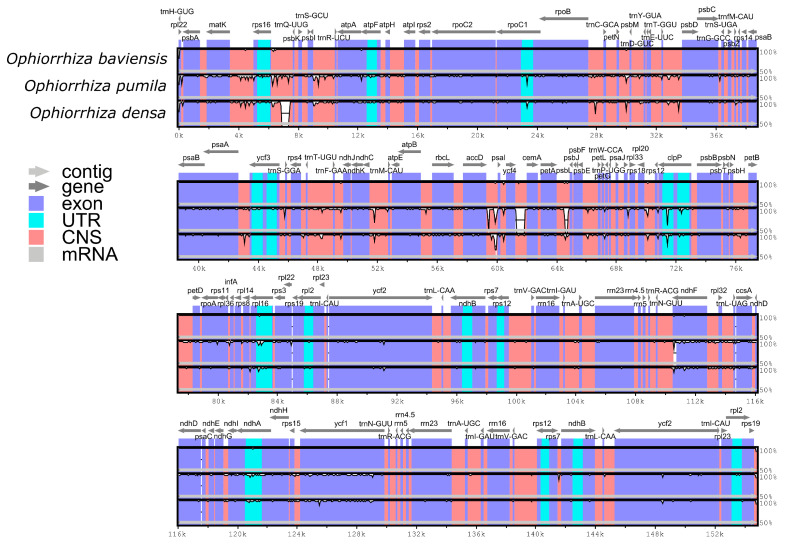
Complete chloroplast genome alignments of the three *Ophiorrhiza* species. The horizontal axis indicates the coordinates within the chloroplast genome. The vertical scale indicates the percent identity within 50–100%. Annotated genes are displayed along the top.

To characterize genomic divergence, the percentage of sequence identity was evaluated for three *Ophiorrhiza* species with the functional annotation of *O. baviensis* Drake as a reference. The comparison using the mVISTA program revealed that the gene organization among the three species was highly similar and there were several regions of sequence variation (Figure 5). The results exhibited a higher frequency of divergence in the LSC and SSC regions than in the IR regions. Moreover, the coding regions of the three cp genomes were observed to be more conserved, whereas a majority of the detected variations were screened in the conserved non-coding sequences (CNS). Among the protein-coding gene sequences, the highly disparate genes consisted of *matK, rpoC2, rpoB, clpP, rpl16, ndhF, ndhA,* and *ycf1.*

**Figure 6:**
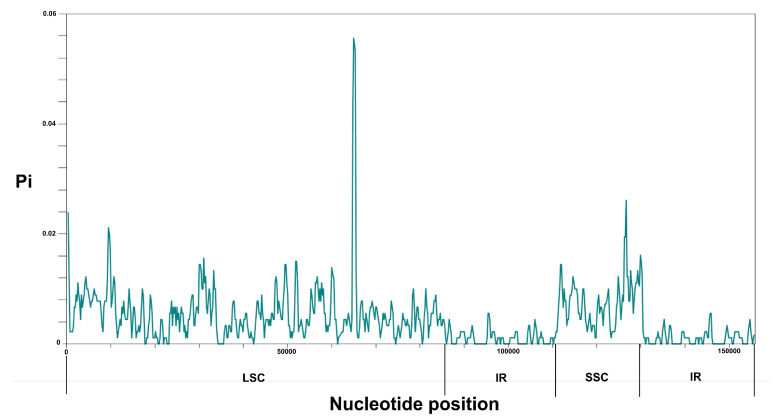
Nucleotide diversity (Pi) values among the three *Ophiorrhiza* species. X-axis: the position in the genome; Y-axis: Pi value. Pi, polymorphism information.

The sliding window analysis indicated that the average polymorphism information (Pi) values of the LSC (Pi = 0.005635) and SSC (Pi = 0.007472) regions were greater than that of the IR (Pi = 0.001285) regions, which showed that most of the variations were located in the LSC and SSC regions (Figure 6). Of the three *Ophiorrhiza* species, the average value of nucleotide diversity (Pi) was 0.00441.

### 3.4. IR Contraction and Expansion in the Chloroplast Genome

**Figure 7:**
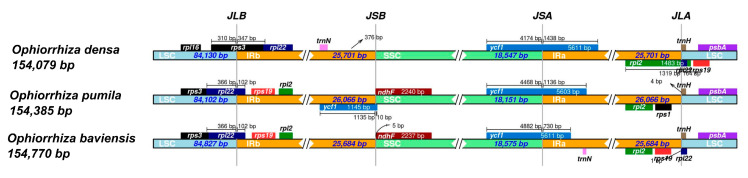
Comparison of LSC, IR, and SSC junction positions among the three *Ophiorrhiza* chloroplast genomes. JLB (junction IRb/LSC), JSB (junction IRb/SSC), JSA (junction IRa/SSC), JLA (junction IRa/LSC).

The IR/LSC and IR/SSC boundaries of three *Ophiorrhiza* cp genomes were compared using the IRscope program. Overall, the results indicated that the region size, gene organization, and gene content showed a high similarity among the cp genome of *O. baviensis* and *O. pumila* (Figure 7). On the other hand, the *O. densa* cp genome showed several variants with the two abovementioned *Ophiorrhiza* species. The size of IR regions ranged from 25,684 bp (*O. baviensis* Drake) to 26,066 bp (*O. pumila*), and the size of IR of *O. densa* was 25,701 bp. The *rpl22* gene was located within the LSC region with a 102 bp overlap with the IRb for *O. baviensis* and *O. pumila,* while *O. densa* showed a 347 bp overlap of the *rps3* gene in this boundary. Apart from *O. densa*, the *ndhF* gene was detected on the boundary of the SSC and IRb region. The border across IRa and SSC was found in the *ycf1* gene with 1438, 1316, and 730 bp tail sections of the gene placed in the IRa of *O. densa, O. pumila*, and *O. baviensis*, respectively (Figure 7). The IRa and LSC boundary showed the presence of the *trnH* gene in the forward strand of all three species and the *rpl22* gene in the reverse strand of *O. baviensis* and *O. densa*. The results of the IR analysis indicated extensive contraction and expansion of the IR regions in the three species.

### 3.5. Phylogenetic Inference

**Figure 8:**
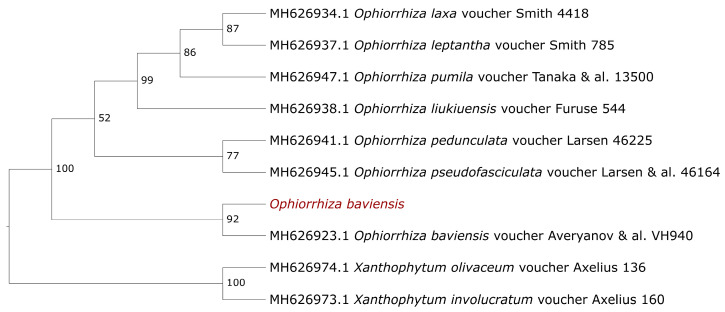
The maximum likelihood phylogenetic trees based on the concatenated sequences between the *rps16* genes and the *trnL-trnF* intergenic spacer. Numbers on the branches indicate bootstrap percentage after 1000 replications in constructing the tree. The species investigated in this study are colored in red.

The number of available sequences of *O. baviensis* on the Genbank databases, especially belonging to the cp genome, is limited (only the *rps16* gene and the *trnL-trnF* intergenic spacer). Therefore, we extracted these sequences from the assembled cp genome and used them to access the phylogenetic relationship of the studied *O. baviensis* at the species level. Figure 8 shows the phylogenetic resolution based on the concatenated sequence between the *rps6* gene and the *trnL-trnF* intergenic spacer with a high bootstrap value of 92% between the studied *O. baviensis* Drake and the reference *O. baviensis* voucher Averyanov & al. VH940 (AAU) (Accession number: MH626923.1). With a bootstrap value of 100%, all eight *Ophiorrhiza* species were grouped separately from the two *Xanthophytum* species as an outgroup. In the case of barcoding among the Rubiaceae family, the combined *rps16-trnL-F* intergenic spacer sequences provided a high capacity for phylogenetic resolution.

## 4. Discussion

Rubiaceae is a family of flowering plants containing 620 genera with approximately 13,500 species over the world, which makes it the fourth-largest angiosperm family. Over 300 cp genomes in the Rubiaceae family have been published in the Genbank database until now, only three of which belong to *Ophiorrhiza*. The genus *Ophiorrhiza* consists of about 200–300 species mainly distributed in humid tropical forests from East India to the Western Pacific, and from South China to Northern Australia [16,17]. Bioactive compounds from this family, such as quinine, emetine, caffeine, and camptothecin are of major pharmaceutical importance; thus, many species in the genus *Ophiorrhiza* are of interest [18]. In the present study, we sequenced and annotated the entire cp genome of a Vietnamese medicinal plant.

Angiosperm cp genomes have a highly conserved gene order and gene content with 127–134 genes found across the chloroplast genomes. The analyzed *O. baviensis* cp genomes demonstrated the typical quadripartite structure and showed the expected size range (~154 kb) for *Ophiorrhiza* species and the conserved gene contents. Our gene annotation results were similar to the genetic properties of angiosperm chloroplast genomes. The number of genes present in the cp genome from *O. baviensis* was 128, of which, 17 genes included one or two introns. In addition, the deletions of the *petB* and *petD* introns were observed in the studied *O. baviensis* cp genome, which also occurred in *O. pumila* species. Introns play an important role in gene expression regulation. Recent research has revealed gene or intron loss in chloroplast genomes [19,20,21], among which *petB* and *petD* intron loss was reported in many angiosperms [22]. 

In addition to two copies of IR regions, 49 small repeats were found to be located within coding and non-coding regions of the *O. baviensis* plastome. The cp genome includes numerous dispersed repeats, which are supposed to be biomarkers of mutational hotspots [23,24]. The repeat number is similar to the data of other species belonging to the Rubiaceae family [25,26]. Repeats are closely related to angiosperm plastome reconstruction and can be assumed as recognition signals of recombination because of their potential to generate secondary structures. In this study, the similar number of repeats in comparison with previous estimates might not demonstrate inter- and intra-specific plastome recombination. In higher plants, SSRs are identified as crucial molecular markers for the investigations of population variation due to their distinct uniparental inheritance, and they are commonly used to evaluate genetic diversity and population structure in evolutionary studies [27,28,29]. In total, 59 SSRs were screened in the *O. baviensis* cp genomes with strong A/T bias. These repeats play a significant role for generating genetic markers in *O. baviensis* species, which may be applied to assess the variation at the intraspecific level in phylogenetic and ecological studies.

Comparative analyses on *O. baviensis* and two available *Ophiorrhiza* cp genomes were implemented to explore the plastome structure in the taxa. The cp genome size of the three *Ophiorrhiza* ranged from 154,079 bp (*O. densa*) to 154,770 bp (*O. baviensis*), the figure for *O. pumila* was 154,385 bp. Gene organization and codon usage patterns exhibited high conservation, which could be applicable for further population genetics and phylogenetic studies. Moreover, the three *Ophiorrhiza* cp genomes were less variable in their coding regions than in their noncoding regions, which is consistent with the common pattern in most angiosperms [30] (Figure 5). Codon usage preference is closely related to gene expression and can affect the level of mRNA and proteins in the genome [31,32,33]. The most prevalent amino acid in the *Ophiorrhiza* was leucine (Leu), which has also been commonly detected in the other angiosperms. The high similarity in codon usage may indicate that these *Ophiorrhiza* species underwent similar environmental pressure through their evolutionary processes. The *Ophiorrhiza* cp genomes indicated that the RSCU values of most codons ending in A/U were greater than 1, which may be caused by a bias toward a high A/T ratio in composition. Additionally, we investigated that the partial sequences of the *ycf1* gene along with five intergenic spacers (IGSs), including *petA-psbJ, trnH-GUG-psbA, trnS-GCU-trnR-UCU, psbM-trnD-GUC,* and *ndhC-trnM-CAU*, had relatively high nucleotide diversity values (Pi > 0.015). These divergence regions could be studied to provide molecular markers for DNA barcoding and phylogenetic research in *Ophiorrhiza*.

While the three plastomes showed an approximate similarity in genome size, the size of the structural regions exhibited significant differences in a detailed comparison of junction sites (Figure 7). The regions of the cp genome frequently undergo length variations during the evolution of terrestrial plants, which leads to the emergence of many boundary features [34]. The expansion and contraction of the boundaries between IRs and the single-copy (SC) regions are the primary causes of the size change in cp genomes and influence the evolution rate of cp genomes [35,36]. Our finding revealed that the boundary-gene set of the *Ophiorrhiza* species included *rpl22, rps19, ndhF, ycf1*, and *trnH*. Several notable gene rearrangements were observed in the *O. densa* plastome; these were the presence of the *rps3* gene at the JLB instead of the *rpl22* gene, the expansion of the *rpl2* gene to the JLA, and the absence of the *rps19* gene in the IR regions. Expansion and contraction, as well as variation, at the junction of the SC–IR regions were characterized, suggesting that gene organization in the IR regions can report the distance between species to some extent. 

The majority of taxonomic levels of plant phylogenetic connections have been demonstrated using complete chloroplast genomes and protein-coding genes [37,38]. The current study provides the phylogeny of the *Ophiorrhiza* genus based on the combined *rps16- trnL-F* intergenic spacer sequences. The previous study of Razafimandimbison and Rydin demonstrated that *O. baviensis* had been resolved as a sister relationship with *O. japonica* and *O. hayatana* [39]. In terms of species classification, the phylogenetic tree based on the concatenation of the *rps16* gene and the *trnL-F* intergenic spacer indicated the close relationship between the studied plant and the *O. baviensis* voucher Averyanov & al. VH940 (AAU) with a high bootstrap value of 92%. This approach showed effectiveness in the classification of the lower taxonomic levels among the Rubiaceae family. Further, the combination of these barcodes can lead to better species classification compared to the results from a single gene [39]. This study will help to clarify the evolutionary position of *O. baviensis* in the *Ophiorrhiza* genus, as well as offering applicable cp genome data for further research into the genesis and diversification of the Rubiaceae family. Overall, our phylogenetic investigation of the *O. baviensis* cp genome was successful in discovering the intrageneric connections within the *Ophiorrhiza* genus. 

## 5. Conclusions

In this study, the first complete chloroplast genome of an *O. baviensis* Drake species from Vietnam was characterized and compared with two other published *Ophiorrhiza* plastomes. The assembly resulted in a whole cp genome of 154,770 bp in size. According to the comparative result, the structure and gene content of three *Ophiorrhiza* cp genomes exhibited a high similarity, and the SC-IR junction analysis revealed the expansion and contraction of IR regions. Additionally, the phylogenetic tree indicated close relationships between our novel cp genome sequence and other *Ophiorrhiza* species. This study provides the potential to employ cp genomes for enhancing species classification and genetic source conservation during further study of the Rubiaceae family.

## Figures and Tables

**Table 1:** Summary of the chloroplast genome of *O. baviensis* Drake species.

Genome Features	*O. baviensis* Drake
Genome size (bp)	154,770 bp
LSC size (bp)	84,826
SSC size (bp)	18,574
IR size (bp)	25,685
GC content (%)	37.6
No. of genes	128
No. of PCGs	87
No. of tRNA	33
No. of rRNA	8

**Table 2:** Gene composition of *O. baviensis* Drake chloroplast genome.

Category of Genes	Group of Genes	Name of Genes
Photosynthesis	Subunits of ATP synthase	*atpA, atpB, atpE, atpFa, atpH, atpI*
	Subunits of NADH-dehydrogenase	*ndhAa, ndhB (×2)a, ndhC, ndhD, ndhE, ndhF, ndhG, ndhH, ndhI, ndhJ, ndhK*
	Subunits of cytochrome b/f complex	*petL, petB, petG, petA, petD, petN*
	Subunits of photosystem I	*psaJ, psaC, psaA, psaI, psaB*
	Subunits of photosystem II	*psbA, psbB, psbC, psbD, psbE, psbF, psbH, psbJ, psbK, psbL, psbM, psbN, psbT, psbZ*
	Subunit of rubisco	*rbcL*
Transcription and translation	Large subunit of ribosome	*rpl14, rpl16a, rpl2 (×2)a, rpl20, rpl22, rpl23 (×2), rpl32, rpl33, rpl36*
	DNA-dependent RNA polymerase	*rpoB, rpoA, rpoC1a, rpoC2*
	Small subunit of ribosomal proteins	*rps11, rps12(×2)a, rps14, rps15, rps16a, rps18, rps19 (×2), rps2, rps3, rps4, rps7 (×2), rps8*
	rRNA genes	*rrn23S (×2), rrn16S (×2), rrn5S (×2), rrn4.5S (×2)*
	tRNA genes	*trnA-UGC (×2)a, trnC-GCA, trnD-GUC, trnE-UUC, trnF-GAA, trnG-GCCa, trnH-GUG, trnI-GAU (×2)a, trnL-CAA (×2), trnL-UAG, trnN-GUU (×2), trnP-UGG, trnQ-UUG, trnR-ACG (×2), trnR-UCU, trnS-GCU, trnS-GGA, trnS-UGA, trnT-GGU, trnT-UGU, trnV-GAC (×2), trnW-CCA, trnY-GUA*
	Translational initiation factor	*infA*
Other genes	Subunit of acetyl-CoA-carboxylase (fatty acid synthesis)	*accD*
	c-type cytochrome synthesis gene	*ccsA*
	Envelope membrane protein (carbon metabolism)	*cemA*
	Protease	*clpPb*
	Maturase (RNA processing)	*matK*
	Conserved open reading frames	*ycf1, ycf2 (×2), ycf3b*

Genes marked with the sign are the gene with a single (a) or double (b) introns and duplicated genes (×2).

**Table 3:** Relative synonymous codon usage (RSCU) for protein-coding genes in *O. baviensis*.

Codon	AA	Frequency	RCSU	Codon	AA	Frequency	RCSU	Codon	AA	Frequency	RCSU
UAA	*	1259	1.22	AUC	I	1205	0.80	CGG	R	420	0.75
UAG	*	825	0.80	AUA	I	1471	0.98	AGA	R	1093	1.94
UGA	*	1004	0.98	AAA	K	2050	1.35	AGG	R	627	1.12
GCU	A	446	1.23	AAG	K	982	0.65	UCU	S	1113	1.39
GCC	A	351	0.97	UUA	L	1040	1.23	UCC	S	982	1.22
GCA	A	401	1.11	UUG	L	1095	1.30	UCA	S	824	1.03
GCG	A	250	0.69	CUU	L	1063	1.26	UCG	S	622	0.77
UGU	C	679	1.20	CUC	L	653	0.77	AGU	S	747	0.93
UGC	C	449	0.80	CUA	L	737	0.87	AGC	S	529	0.66
GAU	D	1012	1.42	CUG	L	480	0.57	ACU	T	668	1.13
GAC	D	413	0.58	AUG	M	856	1.00	ACC	T	651	1.10
GAA	E	1337	1.43	AAU	N	1779	1.38	ACA	T	647	1.09
GAG	E	537	0.57	AAC	N	800	0.62	ACG	T	406	0.68
UUU	F	2212	1.20	CCU	P	611	1.03	GUU	V	784	1.38
UUC	F	1481	0.80	CCC	P	618	1.04	GUC	V	411	0.72
GGU	G	540	0.96	CCA	P	726	1.23	GUA	V	682	1.20
GGC	G	383	0.68	CCG	P	414	0.70	GUG	V	402	0.71
GGA	G	747	1.33	CAA	Q	987	1.40	UGG	W	681	1.00
GGG	G	577	1.03	CAG	Q	420	0.60	UAU	Y	1345	1.36
CAU	H	880	1.36	CGU	R	376	0.67	UAC	Y	637	0.64
CAC	H	414	0.64	CGC	R	280	0.50				
AUU	I	1830	1.22	CGA	R	576	1.02				

* Stop codon.

## Data Availability

This complete chloroplast genome of *O. Baviensis* Drake has been deposited at DDBJ/ENA/GenBank under the accession number OP902221.
